# Metabolic engineering of *Escherichia coli* for efficient production of l-alanyl-l-glutamine

**DOI:** 10.1186/s12934-020-01369-2

**Published:** 2020-06-11

**Authors:** Jiangming Zhu, Wei Yang, Bohua Wang, Qun Liu, Xiaotong Zhong, Quanxiu Gao, Jiezheng Liu, Jianzhong Huang, Baixue Lin, Yong Tao

**Affiliations:** 1 Chinese Academy of Sciences Key Laboratory of Microbial Physiological and Metabolic Engineering, Institute of Microbiology, Chinese Academy of Sciences, Beijing, 100101 China; 2 University of Chinese Academy of Sciences, Beijing, 100049 China; 3 National Engineering Research Center of Industrial Microbiology and Fermentation Technology, College of Life Sciences, Fujian Normal University, Fuzhou, 350117 Fujian People’s Republic of China

**Keywords:** AQ, Metabolic engineering, Whole-cell biocatalysis, l-amino acid α-ligase, Glutamine synthase

## Abstract

**Background:**

l-Alanyl-l-glutamine (AQ) is a functional dipeptide with high water solubility, good thermal stability and high bioavailability. It is widely used in clinical treatment, post-operative rehabilitation, sports health care and other fields. AQ is mainly produced via chemical synthesis which is complicated, time-consuming, labor-intensive, and have a low yield accompanied with the generation of by-products. It is therefore highly desirable to develop an efficient biotechnological process for the industrial production of AQ.

**Results:**

A metabolically engineered *E. coli* strain for AQ production was developed by over-expressing l-amino acid α-ligase (BacD) from *Bacillus subtilis*, and inactivating the peptidases PepA, PepB, PepD, and PepN, as well as the dipeptide transport system Dpp. In order to use the more readily available substrate glutamic acid, a module for glutamine synthesis from glutamic acid was constructed by introducing glutamine synthetase (GlnA). Additionally, we knocked out *glsA*–*glsB* to block the first step in glutamine metabolism, and *glnE*–*glnB* involved in the ATP-dependent addition of AMP/UMP to a subunit of glutamine synthetase, which resulted in increased glutamine supply. Then the glutamine synthesis module was combined with the AQ synthesis module to develop the engineered strain that uses glutamic acid and alanine for AQ production. The expression of BacD and GlnA was further balanced to improve AQ production. Using the final engineered strain p15/AQ10 as a whole-cell biocatalyst, 71.7 mM AQ was produced with a productivity of 3.98 mM/h and conversion rate of 71.7%.

**Conclusion:**

A metabolically engineered strain for AQ production was successfully developed via inactivation of peptidases, screening of BacD, introduction of glutamine synthesis module, and balancing the glutamine and AQ synthesis modules to improve the yield of AQ. This work provides a microbial cell factory for efficient production of AQ with industrial potential.
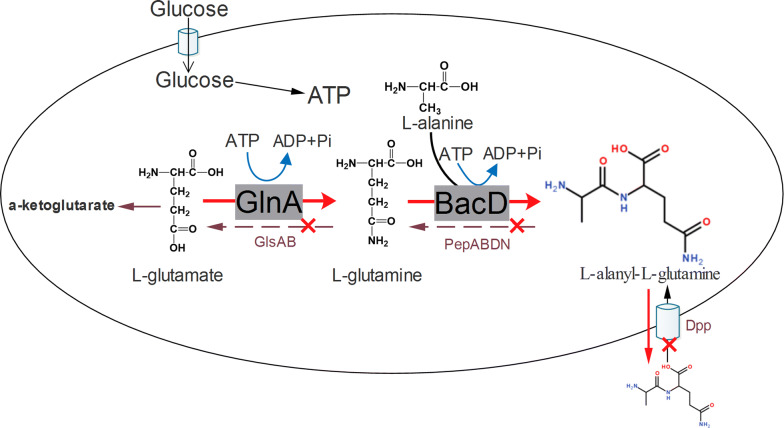

## Background

Glutamine (l-Gln) plays an important role in maintaining intestinal function [1–4], promoting immune function [5, 6], maintaining homeostasis of the internal environment [7] and improving the adaptability of organism to stress [8]. During disease or stress conditions, demand outpaces supply, and glutamine becomes conditionally essential [9]. The supply of exogenous l-glutamine or glutamyl dipeptide is an important nutritional solution to reduce glutamine deficiency in vivo, and can be applied in clinical treatment. However, some characteristics of glutamine such as low solubility in water, easy decomposition and poor thermal stability, as well as production of toxic pyroglutamate during heat sterilization restricted its application in medicine. Otherwise, as a result of its unstable nature, the low yield of glutamine during the purification process is a great challenge [10].

l-Alanyl-l-glutamine (abbreviated as AQ) is a dipeptide of glutamine and alanine, which is more stable and water-soluble than glutamine. AQ is hydrolyzed to release glutamine and alanine in vivo. While it acts as a source of glutamine, AQ has higher bioavailability and a short half-life [11], and does not cause cumulative damage to the body, so it is used as a substitute for glutamine in clinical practice.

The commercial demand for AQ is increasing with the expansion of new applications and the development of new products based on AQ. Chemical and biotechnological methods for AQ production have been developed [12–16]. AQ can be synthesized by chemical condensation of benzyloxycarbonyl-l-alanine and methyl glutamate via activated ester [17] or triphenyl phosphine/hexachloroethane condensation, which used to require complex steps such as amino acid activation, subunit protection, and removing the protective groups. Enzymatic processes for AQ production have been developed using an α-amino acid ester acyltransferase [18, 19]. Tabata and Hashimoto engineered *Escherichia coli* by expressing l-amino acid α-ligase (BacD), which catalyzes the formation of AQ in an ATP-dependent manner, and produced more than 100 mM AQ in 47 h of fermentation [20]. Whole-cell biocatalysis can be used to perform enzyme cascade reaction, improve catalytic efficiency, and simplify the preparation process.

**Fig. 1:**
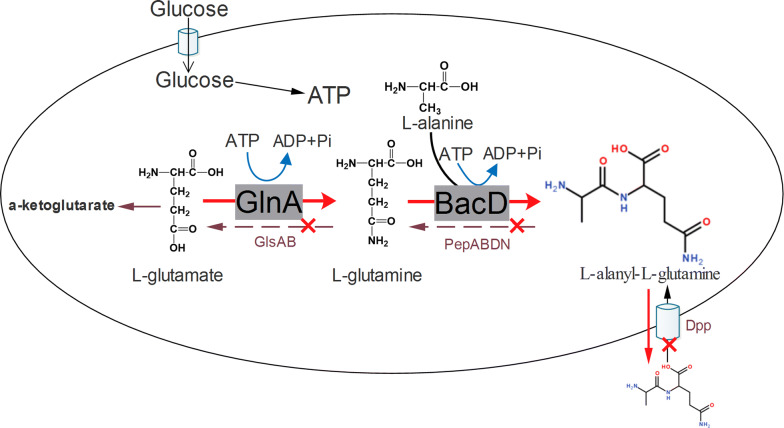
Schematic presentation of the AQ metabolic pathway in engineered *E. coli*. The GlnA and BacD were co-expressed. Discontinued arrows represent the enzymatic activities that have been eliminated. *GlsA* glutaminase 1, *GlsB* glutaminase 2, *GlnE* fused glutamine synthetase deadenylase/adenylyltransferase, *GlnB* nitrogen regulatory protein PII-1, *PepA* aminopeptidase A/I, *PepB* aminopeptidase B, *PepD* peptidase D, *PepN* aminopeptidase N, *Dpp* dipeptide ABC transporter DppABCDF

A metabolically engineered *E. coli* strain for AQ production was developed by over-expressing l-amino acid α-ligase (BacD) from *Bacillus subtilis* and inactivating native peptidases. In order to use a more readily available substrate, the glutamine synthesis module based on introducing glutamine synthetase (GlnA) was constructed and optimized. Then glutamine synthesis module was combined with the AQ synthesis module to use glutamic acid and alanine for AQ production. The protein expression of BacD and GlnA was further balanced to improve the AQ production. Finally, we used the engineered strain to develop a whole-cell biocatalytic process for AQ production (Fig. 1). This work provides an environmentally friendly, highly efficient and cost-effective process for industrial biosynthesis of AQ.

## Results

### Construction of the AQ synthesis module

**Fig. 2:**
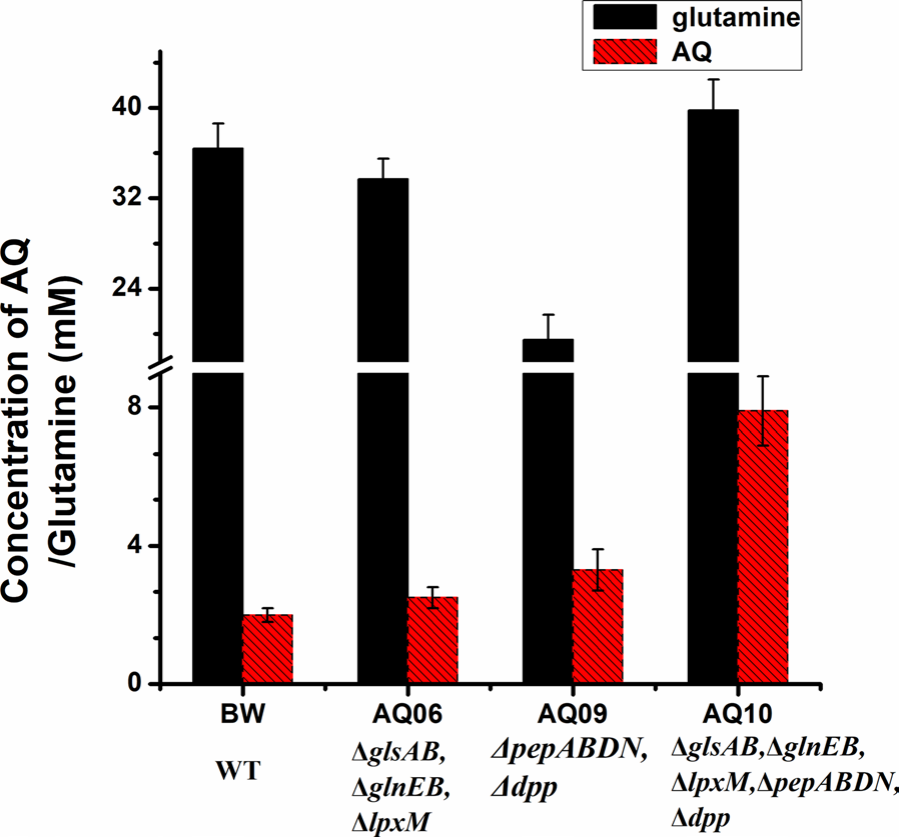
Extracellular titers of AQ produced by engineered strains overexpressing BsBacD. *WT* wild type; BW, AQ06, AQ09, AQ10 were transformed with plasmid p01. The engineered strains were induced and suspended in a reaction mixture containing 50 mM glutamine, 50 mM alanine, and 10 mM magnesium chloride. The bioconversion reactions were performed at 30 °C and 200 rpm for 18 h. Glucose was supplemented at a concentration of 10 mM every 3 h

The *E. coli* strain p01/BW25113, overexpressing BacD from *Bacillus subtilis* (BsBacD) which catalyzes the formation of AQ from alanine and glutamine, was constructed for the production of AQ. Extracellular AQ concentration was measured by HPLC, and 2.0 mM AQ was obtained (Fig. 2).

In *E. coli*, peptidases encoded by *pepA*, *pepB*, *pepD* and *pepN*, have been reported to degrade a broad spectrum of dipeptides [20, 21], and inactivating them might reduce AQ degradation. It was reported that deletion of *dpp*, encoding a dipeptide ABC transporter increased AQ accumulation [20, 22, 23]. By knocking out the genes *pepN*, *pepA*, *pepB*, *pepD* and *dpp,* the degradation of AQ was alleviated. In the starting host BW25113, 20 mM AQ was completely degraded after 3 h, compared to only 1.3 mM in the chassis AQ09 (BW25113*ΔpepN*, *ΔpepA*, *ΔpepB*, *ΔpepD*, *Δdpp*) after 6 h. A whole-cell biocatalysis system with the strain (p01/AQ09) yielded 3.3 mM AQ after 18 h (Fig. 2). These results demonstrated that inactivation of peptidases and the dipeptide transporter Dpp reduced the degradation of AQ, and thus increased AQ production.

### Screening of BacD homologues

**Fig. 3:**
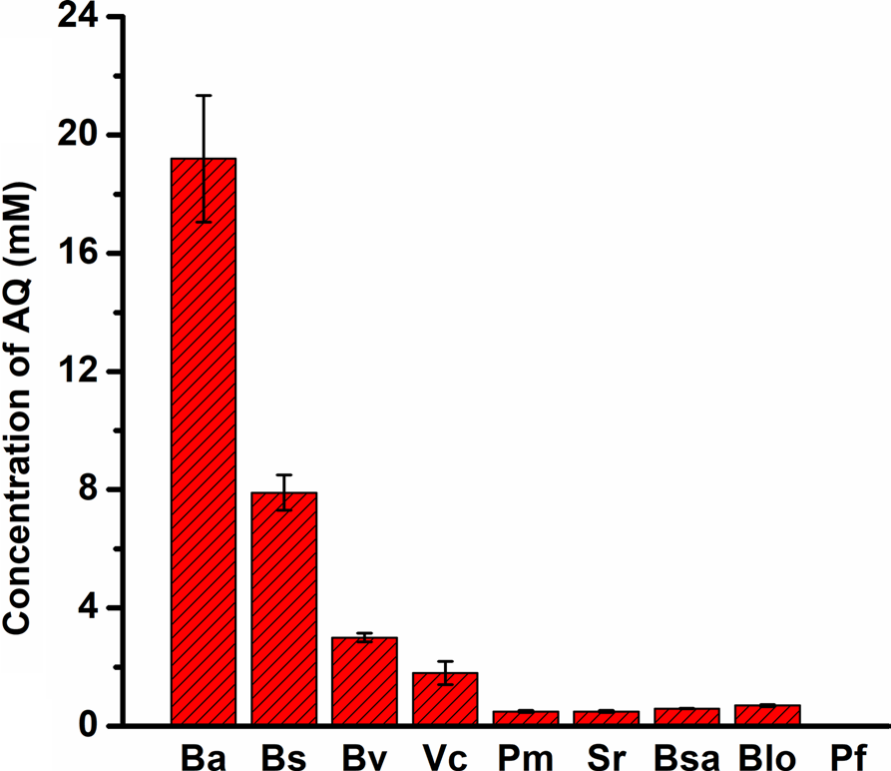
AQ production by engineered strains over-expressing BacD homologs from different species. Ba, BacD from *Bacillus altitudinis*; Bs, BacD from *Bacillus subtilis*; Bv, BacD from *Beta vulgaris*; Vc, BacD from *Vibrio campbellii*; Pm, BacD from *Perkinsus marinus*; Sr, BacD from *Streptomyces rubrolavendulae*; Bsa, BacD from *Bacillus safensis*; Blo, BacD from *Bifidobacterium longum* subsp. *Infantis*; Pf, BacD from *Pseudomonas fluorescens*

BacD is the key enzyme for AQ synthesis. We examined the known sequences annotated as l-amino acid α-ligase (BacD) in the NCBI database. According to the sequences of known or predicted BacD homologs, we selected a set of related sequences from different species, and constructed a phylogenetic tree of the previously reported BacD homologs and those used in this study (Additional file 1: Fig. S1). The genes encoding BacD homologs from different species were codon-optimized by Nanjing Generay (China) and cloned into strain AQ10 (*BW25113*, *∆glnEB, ∆glsAB, ∆lpxM, ∆pepABDN, ∆dpp*). The performance of different BacD proteins was investigated in vivo, using the respective strains as a whole-cell biocatalysts. The result showed that the strain overexpressing BaBacD (from *Bacillus altitudinis*) produced higher amount of AQ (19.2 mM) than strains with other BacD homologs. By comparison, 7.9 mM AQ was obtained using the strain overexpressing BsBacD (from *Bacillus subtilis*) (Fig. 3). Although there was soluble expression of BvBacD (from *Beta vulgaris*), VcBacD (from *Vibrio campbellii*), and SrBacD (from *Streptomyces rubrolavendulae*), only 3.0, 1.8, 0.5 mM AQ was respectively obtained (Fig. 3). BsaBacD (from *Bacillus safensis*), BloBacD (from *Bifidobacterium longum* subsp. *infantis*), PmBacD (from *Perkinsus marinus*), and PfBacD (from *Pseudomonas fluorescens*) were expressed as inclusion bodies (Additional file 1: Fig. S2), and only a low amount of AQ was detected.

### Construction of a glutamine synthesis module

**Fig. 4:**
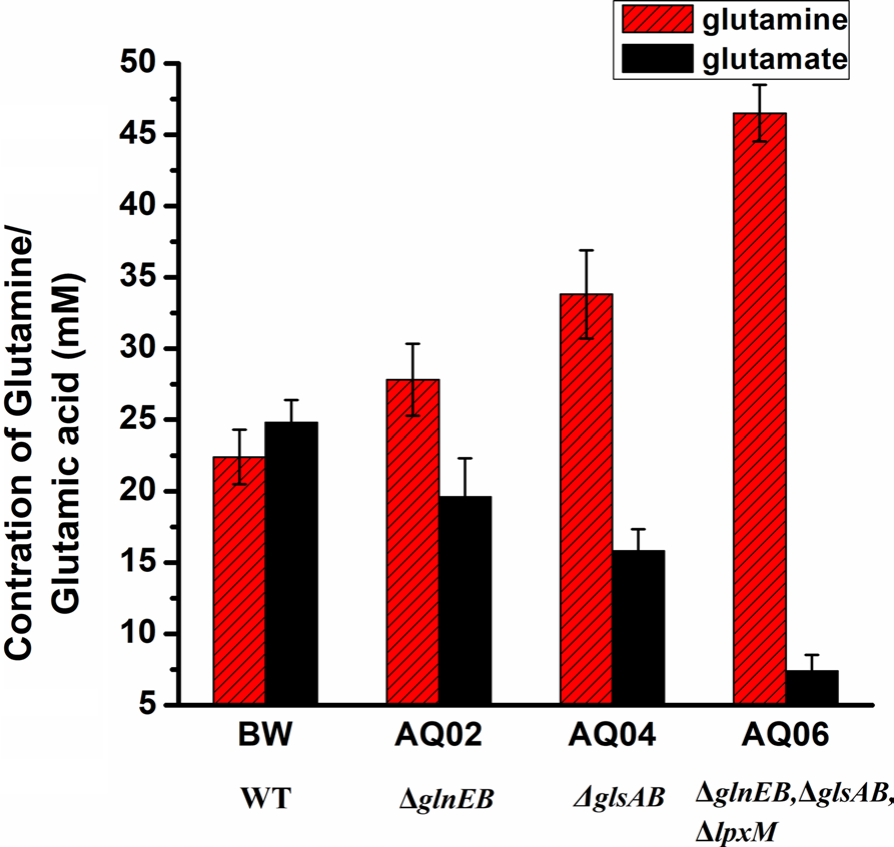
Extracellular glutamine titers produced by engineered strains overexpressing GlnA. *WT* wild type; BW, AQ02, AQ04, and AQ06 were transformed with plasmid p00. The engineered strains were induced and suspended in a reaction mixture containing 50 mM sodium glutamate, 10 mM magnesium chloride, and 100 mM ammonium chloride. The bioconversion reactions were performed at 30 °C and 200 rpm for 18 h. Glucose was supplemented at a concentration of 10 mM every 3 h

To use the more readily available substrate glutamic acid, glutamine synthetase from *Corynebacterium glutamicum* (CgGlnA), which convert glutamic acid to glutamine was cloned into *E. coli*, resulting in the strain p00/BW25113. A final glutamine titer of 22.4 mM was obtained in the whole-cell bioconversion. During growth nitrogen replete growth conditions, glutamine synthetase adenylyltransferase/deadenylase (encoded by gln*E*) interacts with PII-1 protein encoded by *glnB*, which reduces the activity of glutamine synthetase, and *glnE*–*glnB*-deficiency was reported to lead to increased glutamine accumulation [24–26]. Therefore, a *glnE*–*glnB*-deficient strain expressing GlnA (p00/AQ02) was constructed, and a glutamine titer of 27.8 mM was achieved, which was 24.1% higher than that of the strain p00/BW25113 (Fig. 4). In *E. coli*, glutamine was converts into glutamic acid by glutaminases GlsA (encoded by *glsA*) and GlsB (encoded by *glsB*) [27], and further into α-ketoglutarate. Consequently, the *glsA* and *glsB* genes were deleted, resulting in strain AQ04 (*ΔglsAΔglsB*), which was then transformed with the plasmid p00, and the resulting strain p00/AQ04 produced 33.8 mM of glutamine after 18 h of bioconversion. Further, *glnE, glnB, glsA, glsB* and *lpxM* were sequentially knocked out and 46.5 mM glutamine was accumulated by the resulting strain p00/AQ06, with a conversion rate of 93.0% (Fig. 4). Consequently, the strain p00/AQ06 was used for further engineering.

### Combination the AQ and glutamine synthesis modules

To achieve AQ production from glutamic acid and alanine, the AQ and glutamine synthesis modules were combined. The strain AQ10 was obtained by knocking out the genes *pepN*, *pepA*, *pepB*, *pepD*, *dpp*, *glnE, glnB, glsA, glsB* and *lpxM*. In AQ10, the degradation of AQ was alleviated, glutamine catabolism was effectively weakened as well. After introducing BsBacD, the resulting strain p01/AQ10 produced 7.9 mM AQ, which was four times more than the production of the original strain p01/BW25113 (Fig. 2).

**Fig. 5:**
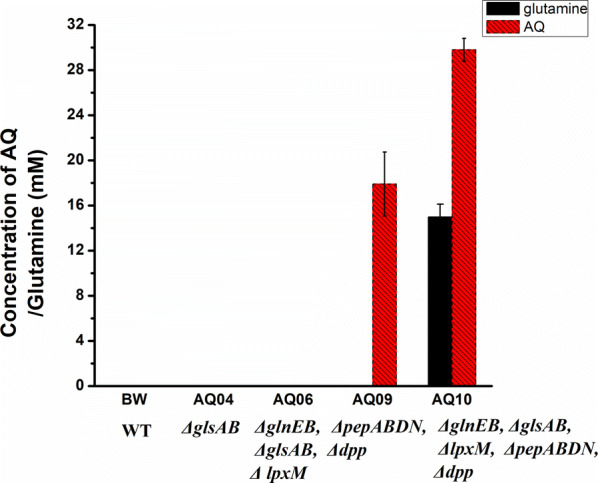
Production of AQ by engineered strains co-expressing CgGlnA and BsBacD. *WT* wild type; BW, AQ02, AQ04, and AQ06 were transformed with plasmid p11. The engineered strains were induced and suspended in a reaction mixture containing 100 mM sodium glutamate, 100 mM alanine, 200 mM ammonium chloride, and 10 mM magnesium chloride. The bioconversion reactions were performed at 30 °C and 200 rpm for 18 h. Glucose was supplemented at a concentration of 10 mM every 3 h

The engineered strains with the plasmid p11 (pYB1s-*CgglnA*-*BsbacD*), co-expressing BsBacD and CgGlnA was used as a whole-cell biocatalyst for AQ production from alanine and glutamic acid. Removal of the peptidases PepA, PepB, PepD, and PepN, together with knocking out the transporter Dpp significantly increased AQ production, and a titer of 17.9 mM was produced by the strain AQ09 harboring p11 (Fig. 5). Due to the deletion of *glnE*–*glnB* and *glsAglsB*, the biosynthesis of glutamine was enhanced, which resulted in increased AQ production, leading to a product titer of 29.8 mM in the strain AQ10 harboring p11 (Fig. 5). Inactivation of peptidases alleviated AQ degradation, and removing the transporter Dpp promoted the efflux of AQ. The results showed that combination of the strategies of peptidases inactivation, knocking out the transporter Dpp, and enhancing the glutamine supply by deletion of *glnE*–*glnB* and *glsA*–*glsB* greatly enhanced AQ production.

### Balance of the two synthesis modules by regulating protein expression

**Fig. 6:**
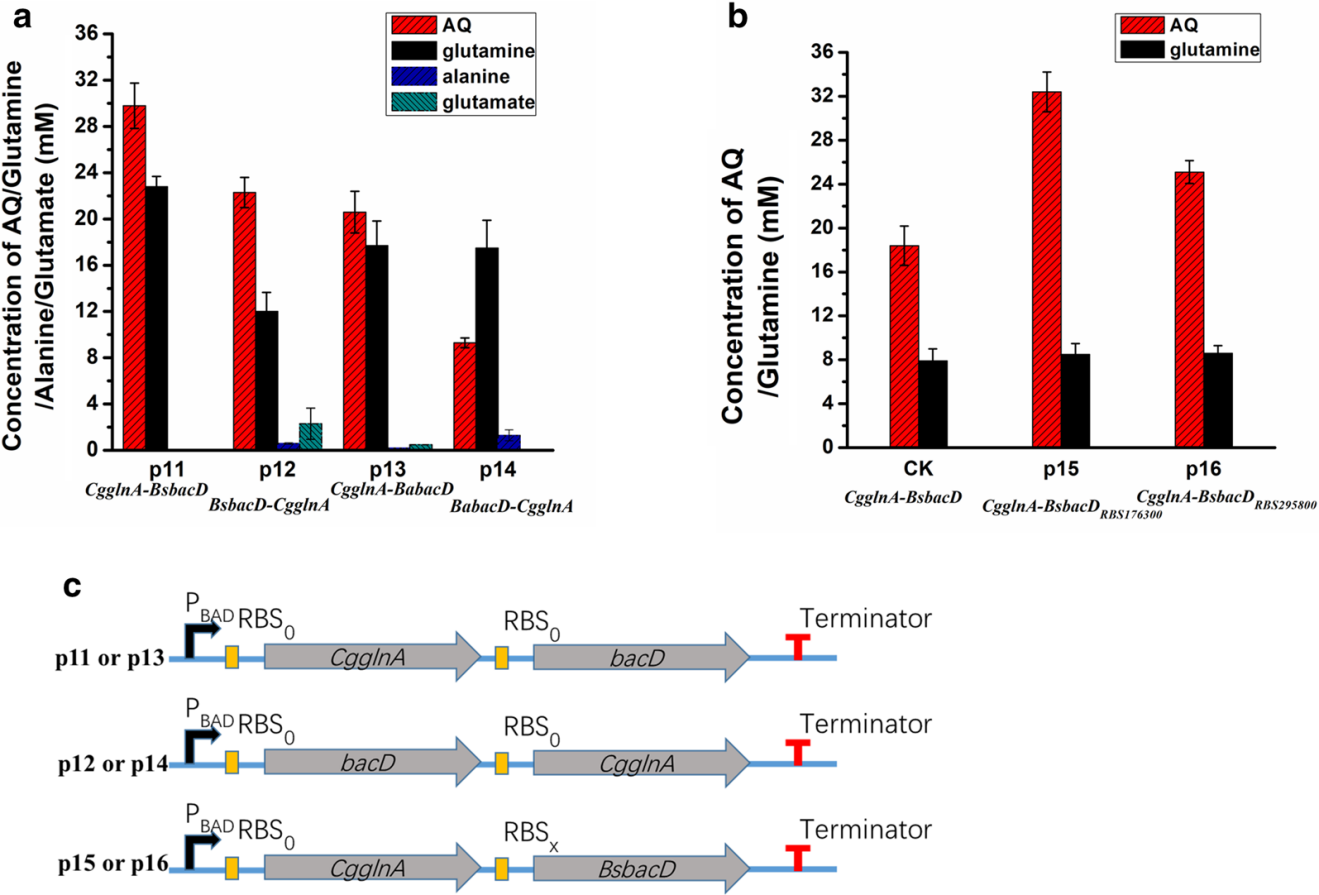
Regulation of the two synthesis modules by balancing the protein expression. **a** Production of AQ in whole-cell bioconversions using engineered strains with *bacD* and *CgglnA* expressed different order. The abbreviations are: p11, pYB1s-*CgglnA*-*BsbacD*; p12, pYB1s-*BsbacD*-*CgglnA*; p13, pYB1s-*CgglnA*-*BabacD*; p14, pYB1s-*BabacD*-*CgglnA*. p11, p12, p13, and p14 were individually introduced into strain AQ10. The engineered strains were induced and suspended in a reaction mixture containing 100 mM sodium glutamate, 100 mM alanine, 200 mM ammonium chloride, and 10 mM magnesium chloride. The bioconversion reactions were performed at 30 °C and 200 rpm for 18 h. **b** Extracellular AQ titer produced by strains with different RBS in the front of *BsbacD* CDS. The reaction was performed in a reaction mixture containing 100 mM sodium glutamate, and 100 mM alanine for 6 h. CK, p11/AQ10. **c** Modular expression of *CgglnA* and *bacD* genes in different order in a *ara*-operon configuration

To balance flux in the two synthesis module for the purpose of increasing AQ production, the expression of BacD and GlnA proteins was studied. To co-express BaBacD or BsBacD with CgGlnA in different order, four plasmids p11 (pYB1s-*CgglnA*-*BsbacD*), p12 (pYB1s-*BsbacD*-*CgglnA*), p13 (pYB1s-*CgglnA*-*BabacD*), and p14 (pYB1s-*BabacD*-*CgglnA*) were constructed (Fig. 6c), and used to individually transform the host AQ10. Either l-amino acid α-ligase or glutamine synthetase was poorly expressed when BaBacD was co-expressed with CgGlnA (Additional file 1: Fig. S3), leading to decreased AQ production. However, when CgGlnA was co-expressed with BsBacD, both proteins were expressed at high levels, and contributed to an increased yield of AQ after 18 h of bioconversion. The AQ titer reached 29.8 mM when *CgglnA* was inserted in front of *BsbacD* (p11/AQ10), compared to 22.3 mM when *CgglnA* was expressed behind *BsbacD* (p12/AQ10) (Fig. 6a). The concentration of the intermediate metabolite, glutamine, in p11/AQ10 (22.8 mM) was higher than in p12/AQ10 (12.0 mM) (Fig. 6a). SDS-PAGE analysis of protein expression (Additional file 1: Fig. S3) and the concentration of glutamine suggested that higher soluble expression of CgGlnA enhanced the supply of glutamine, and increasing the expression of BsBacD might further improve the synthesis of AQ.

In order to enhance the expression of BsBacD, its native RBS was replaced to upregulate the mRNA translation initiation rate in the recombinant strain. The translation rate prediction and design of new RBS was done using RBS Calculator 2.0 [28–30]. The strain p15/AQ10 expressed more BsBacD protein (Additional file 1: Fig. S4), and its AQ production increased by 76.1% compared to p11/AQ10 (Fig. 6b).

### Optimization of the conditions for whole-cell biocatalysis

**Fig. 7:**
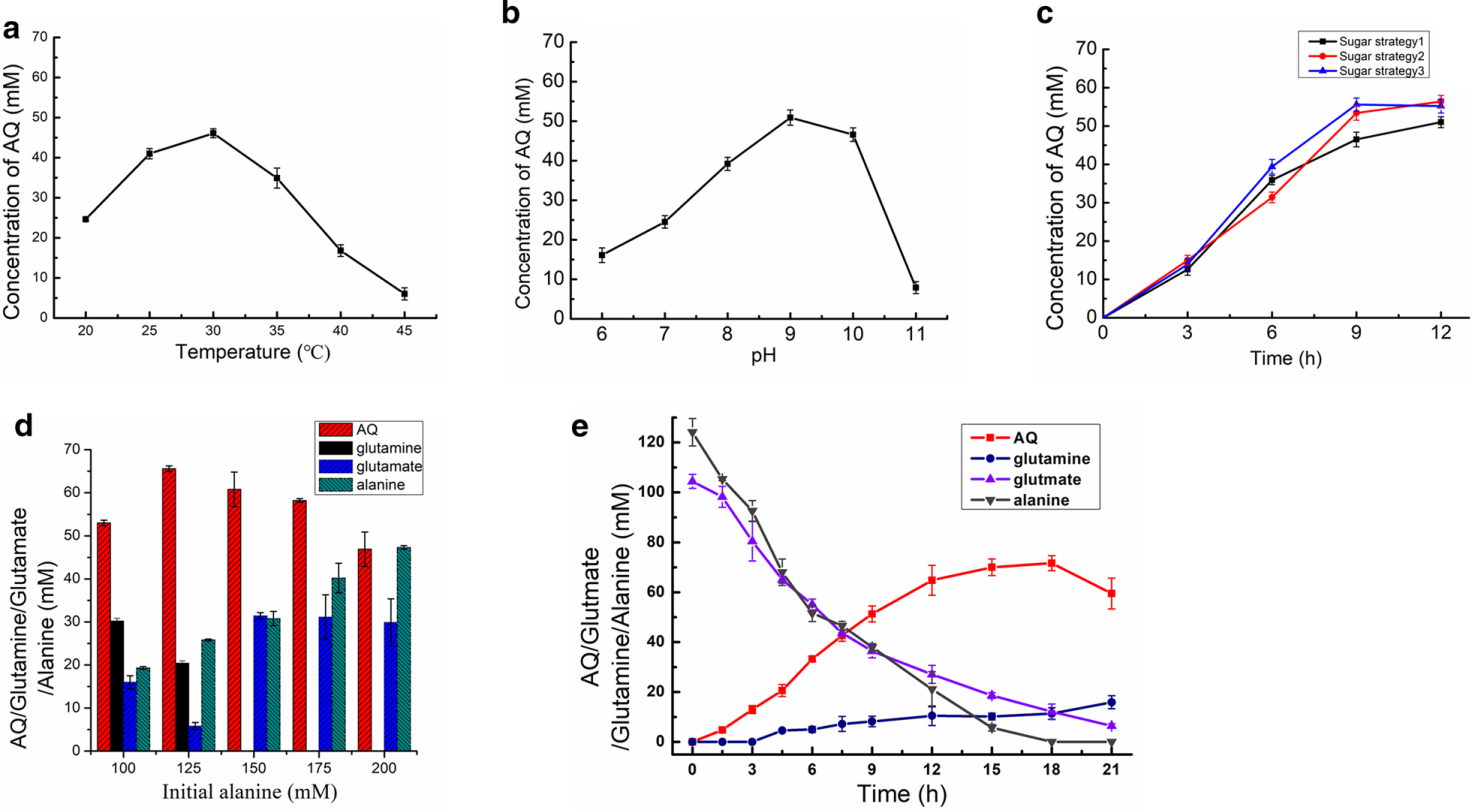
Optimization of the conditions for whole-cell biocatalysis. **a** Effects of pH on AQ production by strain p15/AQ10. **b** Effects of temperature on AQ production by p15/AQ10. **c** Effects of different glucose feeding strategies on AQ production. Feeding strategy: (1) 50 mM glucose was added at once; (2) 10 mM glucose was added every 3 h; (3) 20 mM glucose was added every 3 h. **d** Effects of the initial alanine concentration on AQ production. Initial alanine concentrations of 100–200 mM were combined with a fixed glutamic acid concentration at 100 mM, and the reaction was performed for 12 h. **e** Time profiles of the AQ, glutamine, glutamic acid and alanine concentrations. The bioconversion was performed in a reaction mixture containing 100 mM sodium glutamate, 125 mM alanine, 200 mM ammonium chloride, and 10 mM magnesium chloride at 30 °C and pH 9.0

After successfully constructing an engineered *E. coli* strains for AQ production by metabolic engineering, we investigated its applicability as whole-cell biocatalyst for the biotechnological production of AQ. Bioconversion parameters that affect the activity of the biocatalyst, such as temperature and pH, were investigated. AQ production reached maximal values at 30 °C (Fig. 7a) and pH 9.0 (Fig. 7b). A decreased in pH was observed as the bioconversion proceeded, which affected the biosynthesis of AQ. It should be noted that glucose was supplemented in the reaction mixture to supply ATP for the reactions catalyzed by GlnA and BacD, it was reported that excess glucose can lead to acetate accumulation. Consequently, we measured the concentration of acetate and found that it was accumulated. To alleviate this, different glucose feeding strategies were applied to reduce acetate accumulation in the bioconversion process, including (1) 50 mM glucose at once; (2) 10 mM every 3 h; and (3) 20 mM every 3 h. When a low concentration of glucose (10 mM) was fed every 3 h (Fig. 7c), glucose was fully utilized (Additional file 1: Fig. S5a), and only a small amount of acetic acid accumulated (Additional file 1: Fig. S5b), indicating that 10 mM glucose fed every 3 h matched AQ productivity. The time profiles of the bioconversion indicated that alanine was exhausted first, and the ratio of glutamic acid to alanine was investigated (Fig. 7d). Under feeding with 10 mM glucose every 3 h at 30 °C and pH 9.0, the strain p15/AQ10 produced 71.7 mM AQ, from 100 mM glutamic acid and 125 mM alanine, after 18 h of reaction, corresponding to a productivity of 3.98 mM/h. Moreover, a conversion rate of 71.7% was achieved for glutamic acid, representing a 100% increase compared to the conversion rate before the optimization.

## Discussion

In this study, we constructed a metabolically engineered *E. coli* strain for AQ production by overexpressing l-amino acid α-ligase from *Bacillus subtilis*, knocking out peptidases PepA, PepB, PepD, and PepN, as well as the transporter Dpp, which effectively weakened AQ degradation. To use a more readily available and cheaper substrate, a module for the synthesis of glutamine from glutamic acid was constructed by overexpressing glutamine synthetase from *Corynebacterium glutamicum*. It was reported that the glutaminases GlsA–GlsB convert glutamine to glutamic acid, which is the main pathway of glutamine catabolism, while GlnE interacts with GlnB to induce the adenylation of glutamine synthetase under nitrogen-rich growth, leading to a reduction of the activity of glutamine synthetase. Thus, GlsA, GlsB, GlnE, and GlnB were blocked, which resulted in increased glutamine supply with a 93.0% bioconversion of glutamic acid. Then AQ and glutamine synthesis modules were coupled, and 29.8 mM AQ production was achieved by co-expressing BsBacD and CgGlnA. To balance the flux through two modules, the expression of BacD and GlnA was fine-tuned by modifying the RBS, and AQ production was further increased by 76.1%. Finally, the reaction conditions for the whole-cell biocatalysis were optimized, and 71.7 mM AQ was obtained using strain p15/AQ10 after 18 h of reaction, with productivity of 3.98 mM/h and a conversion rate of 71.7% from glutamic acid to AQ was achieved.

Both fermentation and enzymatic processes for AQ production were reported. We compared the AQ biosynthesis results obtained in this study with previous studies, as shown in Table 1. Enzymatic processes were reported for AQ production from l-alanine methyl ester hydrochloride (AlaOMe) and glutamine using amino acid ester acyltransferase [18, 31, 32]. The substrates used in the process, AlaOMe and glutamine are expensive, making the process not economically feasible. Tabata and Hashimoto reported a fermentative process for AQ production from glucose using a engineered *E. coli* strain co-expressing l-amino acid α-ligase (Lal) and l-alanine dehydrogenase (Ald) in the strain background of JKYPQ3 (*pepA pepB pepD pepN dpp glnE glnB putA*) [20]. The amino acids needed for AQ production were biosynthesized from glucose. However, it was challenging to balance the ratio of glutamine and alanine, which might affect AQ production. Moreover, the synthesis of amino acids was generally tightly regulated. Therefore, AQ accumulation was slow, and the productivity of AQ was low in the fermentation process. In this study, AQ was synthesized from glutamic acid and alanine using whole-cell biocatalytic process. Alanine was added to the reaction mixture as substrate, and glutamine was synthesized from externally added glutamic acid. A very small amount of glucose was used to supply ATP. The concentrations of the substrates (glutamate and alanine) can be easily controlled. The productivity of AQ in this study was 3.98 mM/h which was much higher than previously reported [20].

**Table 1:** Production of AQ using engineered *E. coli*

Strain	Deletion of gene	Overexpression of genes	Process	AQ	References
*E. coli* JKYPQ3/pPE167	*ΔpepAΔpepBΔpepDΔpepNΔdppΔproΔglnBΔglnEΔputA*	l-amino acid α-ligase (Lal/BacD) and l-alanine dehydrogenase (Ald)	Fermentation from glucose	7.4 mM in test tube in 47 h 100 mM (24.7 g/L) in fermenter in 47 h	[20]
*E. brevis* ATCC 14234	None	α-amino acid ester acyltransferase	Enzymatic production by purified enzyme from AlaOMe and Gln	83 mM (18.0 g/L) in 1 h	[31]
*E. coli* SP1/pSaet	*ΔpepD*	α-amino acid ester acyltransferase	Enzymatic production from AlaOMe and Gln	320 mM (69.7 g/L) in 40 min	[32]
*E. coli* OPA	None	α-amino acid ester acyltransferase	Enzymatic production from AlaOMe and Gln	367.9 mM (79.8 g/L) in 20 min	[18]
*E.coli p15/AQ10*	*ΔglsAΔglsBΔlpxMΔglnBΔglnEΔpepAΔpepBΔpepDΔpepNΔdpp*	l-amino acid α-ligase (Lal/BacD) and glutamine synthetase (GlnA)	Whole-cell biocatalytic conversion of glutamic acid and alanine	71.7 mM (14.2 g/L) in test tube in 18 h	This study

It was reported that expression of l-amino acid α-ligase exerted a negative effect on cell growth in the fermentation [20]. In the whole-cell bioconversion, cell growth (the enzyme manufacturing phase) and the AQ production phase were separated. Substrates were converted to AQ by resting cells in the production phase. Therefore, the growth inhibitory effect of l-amino acid α-ligase was reduced.

BacD enzymes have been reported to have insufficient substrate specificity and form by-products, such as different dipeptides or longer oligopeptides [31], which undoubtedly increases the cost of downstream separation and purification of target products. In this study, the extracellular concentrations of Ala–Ala reached 12.4 mM after 18 h of reaction, and no other dipeptides or longer oligopeptides were detected. The concentration of alanine decreased faster than that of glutamic acid, which suggested that the alanine catabolism should be reduced. Further studies are underway to improve the system including screening of BacD homologs with higher enzymatic activity and substrate specificity, enhancing AQ efflux using dipeptide efflux pump, reducing the degradation of alanine by blocking its catabolism, and scale-up of the bioconversion in fermenters.

## Conclusions

In this study, we conducted systematic metabolic engineering of *E. coli* to develop a whole-cell biocatalysis for the synthesis of AQ from glutamic acid and alanine. Inactivation of peptidases and the dipeptide transport system, combined with screening of BacD homologs improved the yield of AQ. The supply of glutamine from glutamic acid was improved by overexpression of glutamine synthetase (GlnA), reduction of glutamine degradation by inactivating of glutaminases (*ΔglsAΔglsB*), and deregulation of glutamine biosynthesis (*ΔglnEΔglnB*). The final engineered *E. coli* strain p15/AQ10 produced 71.7 mM AQ with a conversion rate of 71.7% for glutamic acid. This study offers new opportunities for the bio-industrial production of AQ. The metabolic engineering strategies developed in this study can be applied in the synthesis of other high-value-added dipeptides and oligopeptides.

## Materials and methods

### Construction of plasmids and strains

*Escherichia coli* K12 (BW25113) was used for protein expression. All bacteria strains and plasmids used in this study are listed in Table 2. Target genes (*CgglnA*, *BsbacD*, *BabacD*, *BlabacD*, *BvbacD*, *VcbacD*, *BlabacD*, *PfbacD*, *BlobacD*, *PmbacD*, *BsabacD*, and *SrbacD*) were codon-optimized and synthesized by Generay (Shanghai, China), and then ligated into pYB1a between the Xho I and EcoR I sites via Gibson assembly [33]. Inactivation of genes was conducted using the CRISPR–Cas9 system [34].

**Table 2:** Strains and plasmids used in this study

Strains/plasmids	Characteristics	Source
Strains		
*E. coli* BW25113	*IacI*^*q*^*rrnB*_*T14*_*∆lacZ*_*WJ16*_*hsdR514∆araBAD*_*AH33*_*∆rhaBAD*_*LD78*_	Invitrogen
AQ02	*E. coli* BW25113, *∆glnE, ∆glnB*	This study
AQ04	*E. coli* BW25113, *∆glsA, ∆glsB*	This study
AQ06	*E. coli* BW25113, *∆glnE, ∆glnB, ∆glsA, ∆glsB, ∆lpxM*	This study
AQ09	*E. coli* BW25113, *∆pepA, ∆ pepB, ∆ pepD, ∆ pepN, ∆dpp*	This study
AQ10	*E. coli BW25113*, *∆glnE, ∆glnB, ∆glsA, ∆glsB, ∆lpxM, ∆pepA, ∆ pepB, ∆ pepD, ∆ pepN, ∆dpp*	This study
p11/AQ10	AQ10 expressing p11	This study
p12/AQ10	AQ10 expressing p12	This study
p13/AQ10	AQ10 expressing p13	This study
p14/AQ10	AQ10 expressing p14	This study
p15/AQ10	AQ10 expressing p15	This study
p16/AQ10	AQ10 expressing p16	This study
Plasmids		
pYB1a	P15A origin, pBAD promoter, Amp^R^	Our lab
pYB1s	P15A origin, pBAD promoter, Str^R^	Our lab
p00	*glnA* from *Corynebacterium glutamicum* cloned into pYB1a	This study
p01	*bacD* from *Bacillus subtilis* cloned into pYB1a	This study
p02	*bacD* from *Bacillus altitudinis* cloned into pYB1a	This study
p03	*bacD* from *Beta vulgaris* cloned into pYB1a	This study
p04	*bacD* from *Vibrio campbellii* cloned into pYB1a	This study
p05	*bacD* from *Streptomyces rubrolavendulae* cloned into pYB1a	This study
p06	*bacD* from *Bacillus safensis* cloned into pYB1a	This study
p07	*bacD* from *Bifidobacterium longum subsp. Infantis* cloned into pYB1a	This study
p08	*bacD* from *Brevibacillus laterosporus* cloned into pYB1a	This study
p09	*bacD* from *Perkinsus marinus* cloned into pYB1a	This study
p10	*bacD* from *Pseudomonas fluorescens* cloned into pYB1a	This study
p11	*CgglnA*-*BsbacD* cloned into pYB1s	This study
p12	*BsbacD*-*CgglnA* cloned into pYB1s	This study
p13	*CgglnA*-*BabacD* cloned into pYB1s	This study
p14	*BabacD*-*CgglnA* cloned into pYB1s	This study
p15	*BsbacD* mRNA corresponding translation initiation rate was predicted to be 176300	This study
p16	*BsbacD* mRNA corresponding translation initiation rate was predicted to be 295800	This study

### Culture condition

Strains were grown in LB medium (10 g/L tryptone, 5 g/L yeast extract, 10 g/L NaCl) at 37 °C and 220 rpm. Antibiotics (ampicillin 100 µg/mL, or streptomycin 40 µg/mL) were added as required. For protein expression, auto-inducing ZYM medium (per liter: tryptone 10 g, yeast extract 5 g, glycerol 5 g, glucose 0.5 g, l-arabinose 2 g, Na_2_HPO_4_ 25 mM, KH_2_PO_4_ 25 mM, NH_4_Cl 50 mM, Na_2_SO_4_ 5 mM, MgSO_4_ 2 mM, and trace elements including 0.05 mM FeCl_3_, 0.02 mM CaCl_2_, 0.01 mM MnCl_2_, 0.01 mM ZnSO_4_, and 0.002 mM each of CoCl_2_, NiCl_2_, Na_2_Mo_4_, Na_2_SeO_3_, and H_3_BO_3_) was used [35], and the strains were allowed to auto-induced at 30 °C for 12–16 h.

### Whole‑cell biocatalysis conditions

Cells were harvested after induction by centrifugation at 5000×*g* for 10 min, washed once with 0.85% NaCl solution, and then used for the production of AQ via whole-cell biocatalytic conversion [36]. For AQ synthesis from glutamine and alanine, the conversion system contained 50 mM MOPS (morpholine propane sulfonic acid) buffer pH7.0, 50 mM glutamine, 50 mM alanine, 10 mM magnesium chloride, and 50 mM glucose with a starting OD_600nm_ = 30. The bioconversion reaction was performed at 30 °C and 220 rpm in a test tube. Glucose was supplemented at a concentration of 10 mM every 3 h. For glutamine synthesis, the cells were suspended in 1 mL bioconversion medium containing 50 mM MOPS buffer pH7.0, 50 mM sodium glutamate, 100 mM ammonium chloride, 10 mM magnesium chloride, 50 mM glucose to form a cell suspension with a starting OD_600nm_ = 30. For AQ production from glutamic acid and alanine, the conversion system contained 50 mM MOPS buffer pH9.0, 100 mM sodium glutamate, 100 mM alanine, 100 mM ammonium chloride, 10 mM magnesium chloride, and 50 mM glucose.

In the process of optimizing the whole-cell catalytic conditions, the bioconversion reaction was performed at different temperatures (20–45 °C) and pH values (6.0–11.0). When optimizing the strategy of glucose feeding, three different strategies were investigated: (1) 50 mM glucose at one time; (2) 10 mM glucose was added every 3 h; and (3) 20 mM glucose was added every 3 h. When the concentration of substrate was studied, 100 to 200 mM alanine was added at once, together with 100 mM sodium glutamate.

### Analytical methods

Biomass was estimated by measuring the optical density at 600 nm. Proteins expression was analyzed by SDS-PAGE. The concentrations of glucose and acetate in the supernatant were determined by HPLC on a Bio-Rad Aminex HPX-87H Ion Exclusion column (7.8 × 300 mm; Hercules, CA, USA), with a refractive index detector. Analysis was performed at 55 °C with a mobile phase of 5 mM H_2_SO_4_ at a flow rate of 0.5 mL/min.

The AQ and amino acids including glutamine, glutamic acid, and alanine, were derivatized using 9-fluorenylmethoxy carbonyl chloroformate and measured by HPLC as described by Kazuhiko Tabata [37], with minor modifications as follows. The mobile phase components A and B were acetonitrile and 50 mM sodium acetate, and the gradient program was slightly modified as follows: 0 min, solvent A-solvent B at 10:90; 0 to 20 min, a linear increase in solvent A to A–B at 60:40; 20 to 24 min, a linear increase to A–B at 100:0; 24 to 27 min, held at A–B at 100:0; 27 to 28 min, a linear decrease in solvent B to A–B at 10:90. The column temperature was set at 30 °C, the injection volume was 5 μL, and the flow rate was 0.6 mL/min.

## Supplementary information

**Additional file 1: Fig. S1.** Phylogenetic tree of BacD homologs. **Fig. S2.** SDS-PAGE analysis of different BacD proteins in *E. coli*. **Fig. S3.** SDS-PAGE analysis of strains which co-expressing CgGlnA and BacD. **Fig. S4.** SDS-PAGE analysis of strains by RBS optimization. **Fig. S5.** Concentration of glucose and acetic acid in the conversion.

## Data Availability

All data generated or analyzed during this study are included in this published article and its additional file.
