# Supplementary material for: Metabolic engineering of Escherichia coli for efficient production of l-alanyl-l-glutamine
Source: Microb Cell Fact. 2020 Jun 11;19:129. doi: 10.1186/s12934-020-01369-2 (PMC7291740; doi:10.1186/s12934-020-01369-2)
Supplement: Supplementary file 1 — Additional file 1: Fig. S1. Phylogenetic tree of BacD homologs. Fig. S2. SDS-PAGE analysis of different BacD proteins in E. coli. Fig. S3. SDS-PAGE analysis of strains which co-expressing CgGlnA and BacD. Fig. S4. SDS-PAGE analysis of strains by RBS optimization. Fig. S5. Concentration of glucose and acetic acid in the conversion. [file 12934_2020_1369_MOESM1_ESM.docx]

**Additional file 1**


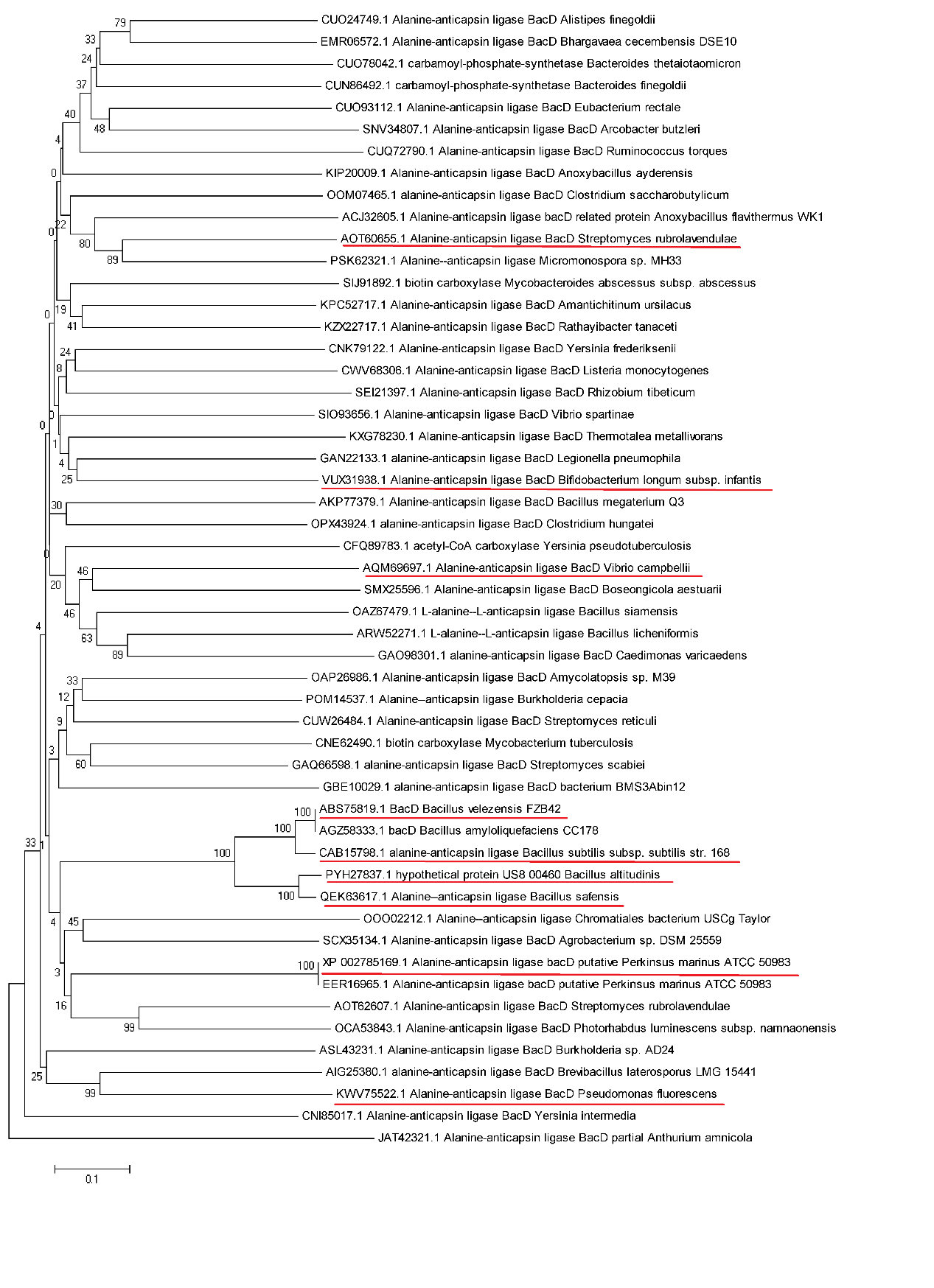


**Fig. S1** Phylogenetic tree of BacD homologs.

The BacD homologs studied in this study were underlined.


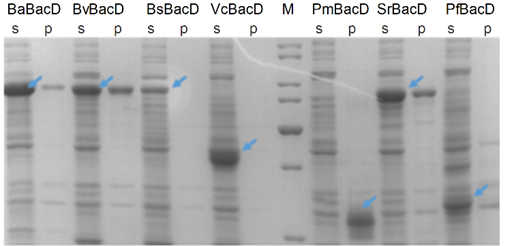


**Fig. S2** SDS-PAGE analysis of BacD homologs in *E.coli*

M: marker, s: supernatant, p: precipitate. BaBacD, BacD from *Bacillus altitudinis*; BsBacD, BacD from *Bacillus subtilis*; BvBacD, BacD from *Beta vulgaris*; VcBacD, BacD from *Vibrio campbellii*; PmBacD, BacD from *Perkinsus marinus*; SrBacD, BacD from *Streptomyces rubrolavendulae*; PfBacD, BacD from *Pseudomonas fluorescens*.


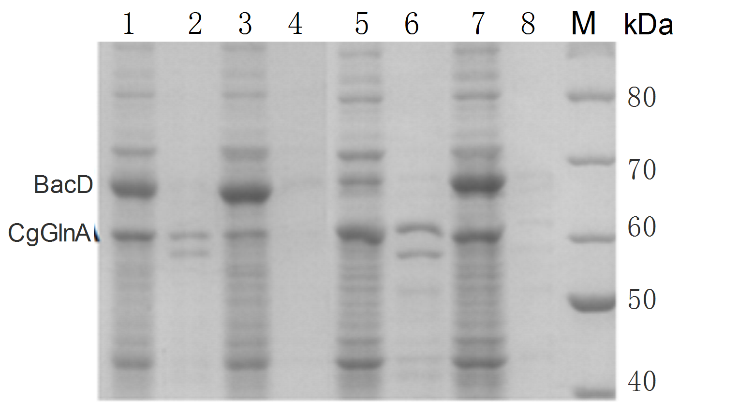


**Fig. S3** SDS-PAGE analysis of strains co-expressing CgGlnA and BacD*.*

M: marker, 1: the supernatant of p11/AQ10, 2: the precipitate of p11/AQ10, 3: the supernatant of p12/AQ10, 4: the precipitate of p12/AQ10, 5: the supernatant of p13/AQ10, 6: the precipitate of p13/AQ10, 7: the supernatant of p14/AQ10, 8: the precipitate of p14/AQ10.


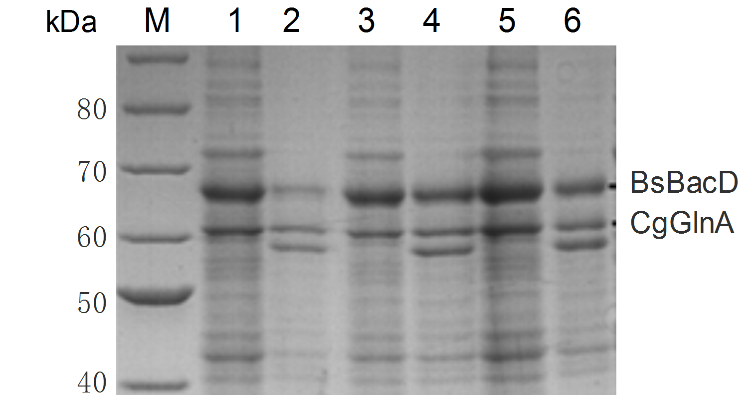


**Fig. S4** SDS-PAGE analysis of strains by RBS optimization.

M: marker, 1: the supernatant of p11/AQ10, 2: the precipitate of p11/AQ10, 3: the supernatant of p15/AQ10, 4: the precipitate of p15/AQ10, 5: the supernatant of p16/AQ10, 6: the precipitate of p16/AQ10.


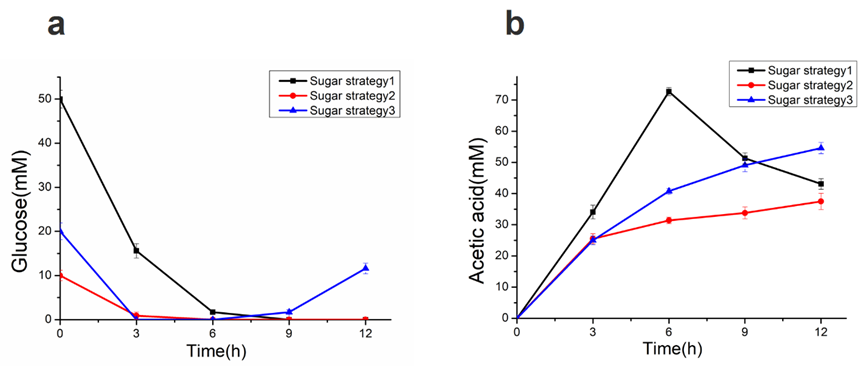


**Fig. S5** Concentration of glucose and acetic acid in the conversion.

**a** Effects of different feeding strategies on residual glucose. **b** Effects of different feeding strategies on acetic acid concentration.
